# Widespread activity of multiple lineages of Usutu virus, western Europe, 2016

**DOI:** 10.2807/1560-7917.ES.2017.22.4.30452

**Published:** 2017-01-26

**Authors:** Daniel Cadar, Renke Lühken, Henk van der Jeugd, Mutien Garigliany, Ute Ziegler, Markus Keller, Jennifer Lahoreau, Lars Lachmann, Norbert Becker, Marja Kik, Bas B Oude Munnink, Stefan Bosch, Egbert Tannich, Annick Linden, Volker Schmidt, Marion P Koopmans, Jolianne Rijks, Daniel Desmecht, Martin H Groschup, Chantal Reusken, Jonas Schmidt-Chanasit

**Affiliations:** 1 Bernhard Nocht Institute for Tropical Medicine, WHO Collaborating Centre for Arbovirus and Haemorrhagic Fever Reference and Research, Hamburg, Germany; 2 These authors contributed equally to this work; 3 Vogeltrekstation – Dutch Centre for Avian Migration and Demography (NIOO-KNAW), Wageningen, the Netherlands; 4 Department of Veterinary Pathology, Faculty of Veterinary Medicine, University of Liège, Liège, Belgium; 5 Friedrich-Loeffler-Institut, Federal Research Institute for Animal Health, Institute of Novel and Emerging Infectious Diseases, Greifswald-Insel Riems, Germany; 6 Parc Animalier de Sainte Croix, Rhodes, France; 7 Nature and Biodiversity Conservation Union (NABU), Berlin, Germany; 8 German Mosquito Control Association (KABSeV), Speyer, Germany; 9 University of Heidelberg, Heidelberg, Germany; 10 Dutch Wildlife Health Centre, Utrecht University, Utrecht, The Netherlands; 11 Erasmus MC, Department of Viroscience, WHO Collaborating Centre for Arbovirus and Haemorrhagic Fever Reference and Research, Rotterdam, The Netherlands; 12 Nature and Biodiversity Conservation Union (NABU), Stuttgart, Germany; 13 German Centre for Infection Research (DZIF), partner site Hamburg-Luebeck-Borstel, Hamburg, Germany; 14 Belgian Wildlife Health Surveillance Network, Department of Infectious and Parasitic Diseases, Faculty of Veterinary Medicine, University of Liège, Liège, Belgium; 15 Clinic for birds and reptiles, University Leipzig, Germany

**Keywords:** Bird, Usutu virus, Germany, Netherlands, France, Belgium, Lineage, Evolution, Epizootic

## Abstract

In the summer of 2016, Belgium, France, Germany and the Netherlands reported widespread Usutu virus (USUV) activity based on live and dead bird surveillance. The causative USUV strains represented four lineages, of which two putative novel lineages were most likely recently introduced into Germany and spread to other western European countries. The spatial extent of the outbreak area corresponded with R_0_ values > 1. The occurrence of the outbreak, the largest USUV epizootic registered so far in Europe, allowed us to gain insight in how a recently introduced arbovirus with potential public health implications can spread and become a resident pathogen in a naïve environment. Understanding the ecological and epidemiological factors that drive the emergence or re-emergence of USUV is critical to develop and implement timely surveillance strategies for adequate preventive and control measures. Public health authorities, blood transfusion services and clinicians in countries where USUV was detected should be aware of the risk of possible USUV infection in humans, including in patients with unexplained encephalitis or other neurological impairments, especially during late summer when mosquito densities peak.

## Introduction

Usutu virus (USUV) is a mosquito-borne flavivirus that was first isolated from a *Culex neavei* mosquito in South Africa in 1959 [1] and emerged for the first time in Europe in 1996 causing deaths among Eurasian blackbirds (*Turdus merula*) in Italy [2]. Since then, USUV has been the causative agent of epizootics and smaller outbreaks among wild and/or captive birds in Austria, Belgium, Czech Republic, France, Germany, Hungary, Spain and Switzerland, with its first emergence in the Netherlands in 2016 [3-7], often resulting in a massive die-off of blackbirds and captive great grey owls *(Strix nebulosa)* [4]. The transmission pattern seems predominantly determined by temperature conditions influencing both the developmental rate of the mosquito vectors and the extrinsic incubation period of the virus in its mosquito hosts i.e. the time required for the development of the virus in its mosquito vector, from the time of uptake of the virus by the mosquito to the time when the mosquito is infective [8].

In 2009, the first human cases of severe encephalitis due to USUV infection were reported from Italy in two immunocompromised persons, demonstrating the zoonotic potential of USUV [9,10]. Recently, a study in the Emilia-Romagna Region in northern Italy, indicated that human USUV infection may not be a sporadic event. In this study, USUV infections in patients with or without neurological impairments occurred more frequently than West Nile virus (WNV) infections in a four-year period [11], highlighting the need for vigilance towards the public health implications of USUV circulation in large parts of Europe.

Here, we describe from a multi-country perspective, patterns of the 2016 USUV epizootic in western Europe and highlight the need for a cross-border analysis in order to gain a proper understanding of USUV spread and evolution and its potential impact on public health.

## Methods

### Bird collection and sampling

Birds from Belgium, France, Germany and the Netherlands were included in this investigation. Dead and live birds were sampled; sampling was performed according to national animal ethics regulations in all countries. Location and date of sampling of live birds as well as the location and date of finding of dead birds were registered.

After signals of USUV outbreaks in the Netherlands and Germany and media reports of blackbirds with neurological illness in Belgium, a dead bird surveillance was started on 3 October 2016 in the Belgian capital, Brussels, and Walloon regions, using information media to request citizens to submit found dead blackbirds. The bird that yielded the Opglabbeek sequence (see phylogenetic tree in the Results) was actively trapped for sampling.

In France, samples of captive birds that had died of unknown causes from 1 August to 20 September 2016 in an animal park in the Lorraine region were submitted to Erasmus Medical Center, Rotterdam, the Netherlands, where USUV infection was determined as cause of death.

Since the first outbreak of USUV in Germany in 2011–12, dead birds sent to the national reference laboratories have been regularly screened for USUV. In addition, active surveillance of living birds has been conducted at selected locations. After the first indication of a new USUV outbreak in Germany at the end of September 2016, German citizens were requested to send in dead birds for USUV screening; this request was made via press releases of involved institutes and subsequent dissemination of the information by different kinds of media.

In the Netherlands, live and dead wild birds and dead captive birds were collected, sampled and analysed as described in [3], in the period from 2 April 2016 to 5 November 2016. In brief, live wild bird samples were obtained through an existing zoonosis-targeted surveillance project; dead wild birds were obtained through the national wildlife disease scanning surveillance programme which relies on post-mortem investigation of carcasses submitted by citizens; dead captive birds were submitted by owners for post-mortem investigation.

### Modelling the basic reproduction number

The daily basic reproduction number (R_0_) is an indicator for the potential spread of an infectious disease through a naïve population. R_0_ was calculated with the temperature-dependent transmission model by Rubel [8] taking various drivers of disease emergence such as host immunity, extrinsic incubation period and vector reproduction rate, into consideration. Daily mean temperature data on a 0.25° regular latitude-longitude grid (E-OBS dataset, January 2009–September 2016) were downloaded from http://www.ecad.eu [12]. For each grid cell, R_0_ values were averaged for the period from June to September 2016. The averaged R_0_ could be interpreted as average number of secondary infections arising from the introduction of a single infected individual into a completely susceptible population during this period [8]. Data analysis and visualisation was conducted with the programme R [13] using the packages plyr [14], lubridate [15], raster [16], colourRamps [17], rworldmap [18], ggplot2 [19] and gridExtra [20].

### Detection and phylogenetic analysis of USUV

Birds found in Belgium were sequenced at the Faculty of Veterinary Medicine, University Liège; in Germany, birds were sequenced at the Bernhard Nocht Institute for Tropical Medicine, Hamburg; French and Dutch birds were sequenced at Erasmus Medical Center (EMC), Rotterdam.

Total RNA from homogenised tissue samples (brain, liver, lung, and heart) was extracted and analysed for the presence of flavivirus RNA by using a modified pan-flavivirus reverse transcription PCR [21]. Direct sequencing of the pan-flavivirus PCR amplicons showed USUV nucleic acid sequences in each positive sample. In Germany (BNI) these were further subjected to PCRs to amplify and sequence a partial region of the USUV non-structural (NS) 5 gene. The complete coding sequences of the Dutch and French bird USUVs were obtained using random-primed 454-based NGS at the EMC in Rotterdam [22] while full genomes of the Belgian bird USUVs were obtained using random-primed sequencing with Ion Torrent PGM technology at UL exactly as described in [23].

To investigate the genetic relationship between the USUV strains responsible for the 2016 European outbreaks and those available in databases, phylogenetic reconstructions were performed using Bayesian Monte Carlo Markov Chain (MCMC) sampling method implemented in BEAST v.1.8.3 [24] and in parallel a maximum likelihood inference in PhyML v3.1 [25] based on partial NS5 gene coding sequences. The MCMC and PhyML trees (data not shown) were reconstructed using Tamura Nei (TN93) model (TN)93 plus Gamma evolutionary model placed by JModelltest2 [26] as the nt substitution model best fit the data. Sequences were aligned using the MAFFT algorithm and then visually inspected in Geneious v9.1.4.

## Results

### Spatial distribution of the epizootics and epidemic modelling

In 2016, there were a total of 17 live and 147 dead USUV-positive birds reported in the four countries. In Germany, besides recurrent circulation in known affected areas, USUV expanded its geographical distribution. In the Netherlands, USUV RNA was detected for the first time in two healthy blackbirds in the beginning of April 2016 [3].

**Figure 1:**
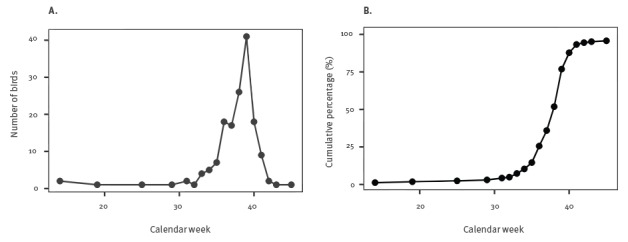
Number (panel A) and cumulative percentage (panel B) of outbreak-related USUV-positive live and dead birds, western Europe^a^, 2016 (n=164)^b^

Since early August (week 31), there was an increasing number of reported disease-associated mortality in blackbirds and captive great grey owls from Belgium, France, Germany and the Netherlands, that peaked in September (weeks 35–39) (Figure 1).

USUV: Usutu virus.

^a^ Belgium, France, Germany and the Netherlands.

^b^ No collection date information was available for seven of the 164 USUV-positive birds which were excluded from this figure.

**Figure 2:**
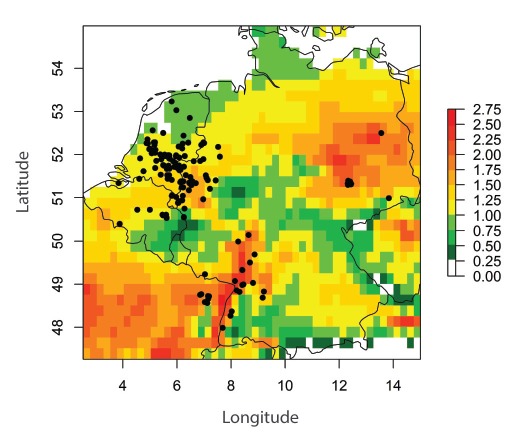
Mean daily basic reproduction number and distribution of outbreak-related USUV-positive birds, western Europe^a^, 2016 (n=164 birds)

Of the 17 live and 147 dead USUV-positive birds reported in 2016, 120 were detected in the tristate area of Belgium, Germany and the Netherlands. The spatial distribution of the majority of positive cases in 2016 fell in an area with a mean basic reproduction number larger than one (R_0_ > 1) (Figure 2).

USUV: Usutu virus.

^a^ Belgium, France, Germany and the Netherlands.

The map represents the spatial distribution of the potential risk for a USUV outbreak between June and September 2016 and the laboratory-confirmed USUV-positive live and dead birds (the black dots) in western Europe. The temperature-dependent transmission model by Rubel et al. [8] was used on the base of daily mean temperature data on a 0.25° regular latitude-longitude grid downloaded from http://www.ecad.eu.

**Figure 3:**
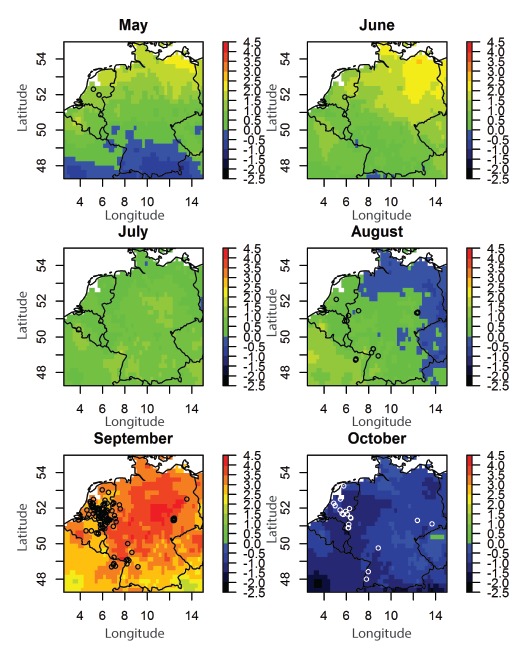
Monthly temperature anomalies and distribution of outbreak-related USUV-positive birds, western Europe^a^, 2016 (n=164)^b^

This R_0_ was driven by extraordinary high temperatures during September 2016, with values exceeding the long-term mean (1986–2015) by more than 3 °C (E-OBS dataset, http://www.ecad.eu) (Figure 3).

USUV: Usutu virus.

^a^ Belgium, France, Germany and the Netherlands.

^b^ No collection date information was available for seven of the 164 USUV-positive birds which were excluded from this figure.

The maps represent the monthly temperature anomalies, i.e. deviation from the 30-year mean temperatures (E-OBS dataset, http://www.ecad.eu), in degree Celsius for the months from May to October 2016 and the laboratory-confirmed USUV-positive live and dead birds (black or white circles) in western Europe, per month. Circles for the month of May include two USUV-positive birds from April 2016 and circles for the month of October include one USUV-positive bird from November 2016.

### Genetic characterisation of epizootic strains

**Figure 4:**
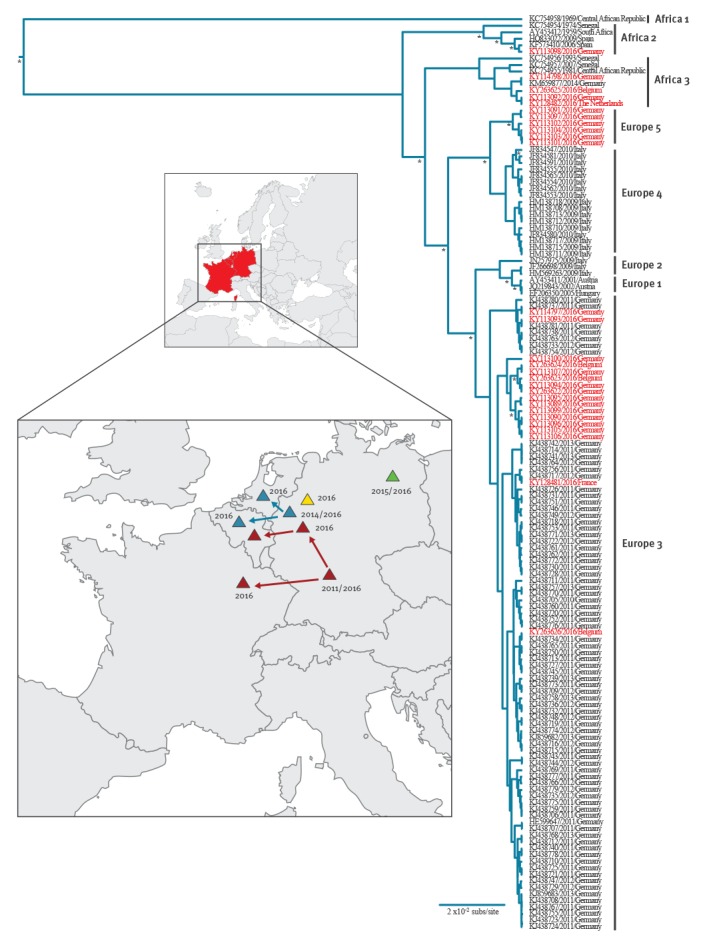
Phylogenetic tree of USUV variants responsible for outbreaks in captive and wild birds and the possible origin and spread pattern, western Europe^a^, 2016

NS: non-structural; nt: nucleotide; USUV: Usutu virus.

^a^ Belgium, France, Germany and the Netherlands.

In total, 28 positive samples (22 from Germany, 4 from Belgium, 1 from the Netherlands, 1 from France) were characterised based on partial sequences of the NS5 coding region (Figure 4).

The tree is based on the partial NS5 gene and it shows placement of the USUV variants that were detected during the ongoing outbreaks in comparison to all available USUV sequences from GenBank. To improve visualisation, phylogenetic positions of the USUV detected in 2016 are bold and red. Taxon information includes the GenBank accession number, isolation/detection year and country in which the virus was isolated/detected. Scale bar indicates mean number of nt substitutions per site. The small map indicates the European countries which have reported USUV outbreaks (red) in 2016, while the magnified map shows the possible origin of different USUV lineages (coloured triangles) involved in the recent European outbreaks. Arrows indicate the likely trend of spatial diffusion pattern of the USUV (coloured based on USUV lineages).

Previous studies showed that this partial NS5 sequence exhibits a phylogenetic signal similar to the complete genome [5,27-29] allowing a rapid characterisation of the circulating virus strains. The 2016 USUV strains represented four lineages (Figure 4). The viruses detected in Belgium, France and the Netherlands clustered with viruses that previously circulated in mosquito vectors, wild birds and/or bats in Germany between 2011 and 2014 [5,6,27,29].

In Germany, a putative novel USUV lineage, called Europe 5, was identified and this was constituted of strains found in birds in west-central North Rhine-Westphalia while lineage Europe 3 USUV emerged outside the previously known endemic areas [6]. The Africa 2 strain that killed two great grey owls in the Berlin Zoo in 2015 [28] was found in 2016 outside the zoo, in a blackbird.

## Discussion

Since the first large outbreaks in the 2000s [7], USUV has become a potential public health concern given the increasing number of reported human infections [9-11,30]. Arbovirus surveillance programmes based on birds and mosquitoes have been conducted in western Europe in recent years and allowed us to elucidate the possible origin, pattern of spatial dynamics, and eco-epidemiological factors that contributed to the 2016 epizootic. It can be speculated that the USUV lineages detected in Belgium, France and the Netherlands were most likely imported from Germany via infected semi-resident wild birds. Introduction via active/passive mosquito dispersal is another possible scenario that was contemplated for WNV, a closely related flavivirus with a similar avian-mosquito life-cycle, as well [31-33]. However, independent long-distance introductions via migratory birds cannot be excluded and geo-phylogenetic analysis of USUV genomes in more birds with a wider geographic coverage, especially in France and the Netherlands, will increase our understanding of the dispersal of USUV across Europe.

The presence of a Europe 3 lineage strain in France and an Africa 3 strain in the Netherlands could each represent a single introduction event with Germany as possible source (Figure 4). In contrast, the USUV epizootic in Belgium was linked to both lineage Africa 3 and Europe 3, indicative for at least two distinct introductions.

The USUV Africa 2 strain found in Berlin seemed to be restricted to this city, thereby supporting the observation that the adaptation of USUV to naïve vector and host populations can lead to the emergence of local virus variants [5]. The geographically distinct lineages occurring in Europe are separated from each other by barriers such as climate, vegetation, different host species, and other unknown ecological conditions [5]. Nevertheless, the synchronous emergence of different USUV lineages in western Europe and their co-circulation in the same regions indicate similar basic ecological parameters driving the transmission of the different lineages involved in the recent outbreak.

The high activity of USUV in the late summer-beginning of autumn of 2016 could be linked to temperature anomalies in September, i.e. significant positive deviation from the 30-year mean temperatures, which will have shortened the extrinsic incubation period, and caused an increase in the vector abundance and therefore the associated vector-host contact rate, at the same time [8]. Based on the known epidemiology of USUV in Europe and given the expected increasing temperatures due to climate change, there could be a risk that the already established USUV loci will expand and further large outbreaks will occur in naïve regions resulting in an increased infection pressure on humans.

The current USUV outbreak exhibited similar patterns to the outbreak of the closely related WNV lineage 2 in central Europe in 2008–2009 when, after a few years of limited local circulation, the virus subsequently spread to Balkan states and northern Greece, where it caused a neuroinvasive disease outbreak among humans with 197 cases [34,35].

Early detection of enzootic circulation based on mosquito and avian surveillance can ensure timely implementation of prevention and control measures. Data from the Dutch USUV outbreak showed that signalling based on live bird surveillance can precede signals from dead bird surveillance up to five months [3]. Enhanced surveillance and monitoring of the densities and infection level of the vector should support the timeliness of bird surveillance. Based on the availability of near real-time temperature data, surveillance sites and time periods with high risk for virus activity can be determined by continuing spatial-temporal analysis. Our findings in the context of what is known about the USUV ecology, emphasise the need for a transboundary strengthening of collaboration and coordination across different research, veterinary and public health sectors, for an effective control and implementation of specific preventive measures.

The adaptation of USUV to naïve vector and vertebrate host populations by introduction/reintroduction of the virus and migratory bird flyways are considered key determinants in the spatial dispersal and establishment of USUV. Thus, multiple complete genome analyses are clearly necessary to fully understand the impact of ecological/immunological/virological factors on USUV epidemiology and evolution of different virus lineages [5]. The recent observations on human USUV infections in northern Italy [11] and the continuous geographic expansion of USUV in Europe should raise awareness among physicians to include USUV in the differential diagnosis of encephalitis cases of unknown aetiology, and among policymakers to address putative issues with blood safety and wildlife conservation alike.
